# A new era for *Inflammation and Regeneration*

**DOI:** 10.1186/s41232-016-0003-8

**Published:** 2016-04-25

**Authors:** Hideyuki Okano

**Affiliations:** Department of Physiology, Keio University School of Medicine, 35 Shinanomachi, Tokyo, Shinjuku-ku 160-8582 Japan

It is my great pleasure to announce the launch of new alliance between our journal, *Inflammation and Regeneration*, and an international publisher, BioMed Central. *Inflammation and Regeneration* is the official journal of the Japanese Society of Inflammation and Regeneration. *Inflammation and Regeneration* provides a forum which covers a wide range of scientific topics in the research field of inflammation and basic and clinical regenerative medicine, such as basic biology of somatic stem cells [1], pluripotency [2], direct reprogramming [3], cell therapy using iPS cell technology [4], disease modeling with iPS cell technology [5], immunoregulation by mesenchymal stromal cells [6], immunoregulation in regenerative medicine [7], tissue homeostasis in health and disease [8], tissue engineering [9], interaction between gut microbiota and host immune cells [10], dendritic cells [11], vascular biology [12], cancer immunology [13], regulatory T cells and immunomodulation [14], intestinal immunity and inflammation [15], inflammation and oxidative stress [16], cellular and molecular bases for fibrotic diseases [17], and new therapeutics of autoimmune disorders [18]. As are the cases for these topics, inflammation research and regenerative medicine are tightly linked research areas in life sciences and their significance in translational research is rapidly increasing. To promote globalization of the journal, inclusion within PubMed Central, and early acquisition of an impact factor, we have decided to strengthen the organization of the journal by establishing this alliance with an international, reliable publisher. Dedicated to open research, BioMed Central publishes a large portfolio of peer-reviewed, open access journals in biology, clinical medicine, and health. From now on, I, as the Editor-in-Chief, encourage the submission of manuscripts whose scope bridges the fields of inflammation and regeneration.
